# Assessing the efficacy of thermotherapy combined with chemotherapy or cryotherapy for the eradication of grapevine leafroll-associated virus 3

**DOI:** 10.3389/fpls.2025.1693493

**Published:** 2026-01-05

**Authors:** Solomon Peter Wante, Kar Mun Chooi, Ranjith Pathirana, Annabel Whibley, Ellie L. Bradley, Yusmiati Liau, Bhanupratap Reddy Vanga, Amy M. Hill, Darrell Lizamore

**Affiliations:** 1 Grapevine Improvement Laboratory, Bragato Research Institute, Lincoln, New Zealand; 2 Plant Pathogen Environment, New Zealand Institute for Bioeconomy Science Limited, Mt Albert, Auckland, New Zealand; 3 School of Agriculture, Food and Wine, University of Adelaide, Adelaide, SA, Australia

**Keywords:** virus elimination, ribavirin, oseltamivir, tissue culture, grapevine, leafroll virus

## Abstract

**Introduction:**

Grapevine leafroll-associated virus 3 (GLRaV-3) poses a significant threat to viticulture and is the major virus pathogen in New Zealand. The presence of the virus is therefore undesirable within the New Zealand Winegrowers National Vine Collection, which serves as a repository of diverse and valuable grapevine genotypes.

**Methods:**

This study evaluated the effectiveness of *in vitro* virus eradication protocols, specifically thermotherapy combined with chemotherapy (ribavirin or oseltamivir) or cryotherapy, for eliminating GLRaV-3 from infected grapevine cultivars. Virus presence was initially confirmed using enzyme-linked immunosorbent assay (ELISA) and high-throughput sequencing (HTS). Following treatment, plantlets were screened using reverse transcription quantitative polymerase chain reaction (RT-qPCR) for GLRaV-3 detection.

**Results:**

Thermotherapy followed by cryotherapy achieved the highest virus elimination rates across cultivars, including complete eradication in Sauvignon Blanc 217, Chenin Blanc, and Riesling Gm 239. Oseltamivir chemotherapy combined with thermotherapy showed higher elimination rates than ribavirin-based treatments, with complete success in Ehrenfelser and Golden Chasselas.

**Discussion:**

Cultivar-specific responses emphasise the need to optimise treatment protocols to achieve broad efficacy across diverse cultivars. After virus elimination, tissue-cultured material could be maintained *in vitro* or by cryopreservation for long-term conservation. These findings provide a scalable strategy for restoring high-health status to grapevine cultivars within germplasm repositories, thereby supporting the long-term sustainability of viticulture.

## Introduction

Grapevine (*Vitis vinifera* L.) is one of the oldest cultivated crops, and towards the end of last century, 10,000 to 14,000 cultivars were estimated to have been conserved in global germplasm collections (Alleweldt, 1994). Subsequent advances in molecular diagnostics have greatly expanded this number, with over 32,000 accessions now reported in grapevine germplasm collections across Europe alone (Maul and This, 2008; Magon et al., 2023; Kotni et al., 2023). Despite its global importance as a major horticultural crop, grapevine productivity and longevity are severely reduced by viral infections (Maliogka et al., 2015; Chooi et al., 2022; National Academies of Sciences, Engineering, and Medicine, 2025). Grapevine’s perennial growth habit, clonal propagation, and exchanges of plant material exacerbate the persistence and spread of grapevine viruses.

In New Zealand, the New Zealand Winegrowers National Vine Collection (NVC) is maintained as a vineyard planting at Lincoln University and serves as a critical repository of grapevine genetic diversity. The NVC is organised into two distinct sections: a high-health collection, comprising virus-indexed genotypes considered free of major pathogens, and a low-health collection, containing approximately 400 genotypes exhibiting symptoms associated with multiple viral infections. HTS of cultivars such as Chenin Blanc and Sauvignon Blanc in the low-health section confirmed the presence of grapevine leafroll-associated virus 3 (GLRaV-3) alongside multiple other viral pathogens (Chooi et al., 2024). This contamination significantly limits the suitability of these vines for research, breeding, and industry propagation.

The persistence of viral infections in grapevines is of particular concern, as viruses such as GLRaV-3, a member of the Closteroviridae family, reduce vine vigour, productivity, and longevity (Fuchs, 2020). In New Zealand, GLRaV-3 is the predominant and most economically significant RNA virus associated with grapevine leafroll disease (Bell et al., 2021; Diaz-Lara et al., 2018; Chooi et al., 2022). Among the vines of the NVC, insects particularly mealybugs, may continue to act as vectors for the spread of GLRaV-3 and other viruses, further compromising vine health and threatening the integrity of the collection. These findings highlight the critical importance of maintaining a library of virus-free vines, including duplicates of diverse genotypes currently represented in the low-health collection. Effective management strategies that can reliably eliminate GLRaV-3 from germplasm repositories are, therefore, essential.

Virus sanitation techniques, including thermotherapy, chemotherapy, and cryotherapy, have been effectively employed to eliminate a broad spectrum of grapevine viruses and viroids, including GLRaV-3 (Panattoni et al., 2013; Bettoni et al., 2016; Bi et al., 2018; Turcsan et al., 2020). However, few studies have systematically compared these techniques across diverse genotypes within a single germplasm resource under uniform diagnostic and culture conditions. Identifying a protocol that is suitable for deployment across diverse genotypes is an important first step in rehabilitating a virus-contaminated collection, particularly where the importation of new material is not feasible.

In this context, *in vitro* culture systems are especially valuable. Beyond their use for efficient clonal propagation, crop improvement (including gene editing), conservation, and targeted metabolite production, *in vitro* culture systems also enable virus elimination from infected clones (Panattoni et al., 2013; Wang et al., 2018; Magyar-Tábori et al., 2021; Wante et al., 2022; Wijerathna-Yapa et al., 2025). Together, these methods underscore the robustness and versatility of *in vitro* approaches for controlled treatments, ranging from chemical elicitation and abiotic stress to virus-sanitation workflows conducted entirely under aseptic conditions or via brief non-aseptic exposure followed by re-entry into sterile culture (Panattoni et al., 2013; Wante et al., 2022). Mechanistically, thermotherapy-based sanitation protocols exploit the apical dome’s limited vascular connectivity and the lower thermal tolerance of most plant viruses relative to host meristematic cells. Plants are typically maintained at 35 to 42 °C for four to six weeks, conditions that restrict virus movement towards the apex, suppress virus replication, and promote viral RNA degradation, resulting in meristematic tissues with reduced viral loads (Spiegel et al., 1993; Wang et al., 2018). Thermotherapy also boosts host antiviral defence by up-regulating key components of RNA silencing systems, increasing the accumulation of virus-derived small interfering RNAs (vsiRNAs), and modulating miRNA-mediated regulation of genes involved in disease defence and hormone signalling (Liu et al., 2015, 2016; Szittya et al., 2003; Wang et al., 2008, 2016). Together, restricted virus movement, diminished replication, and enhanced RNA silencing facilitate the recovery of virus-free meristems and the regeneration of healthy plantlets.

Additional measures, including supplementing culture media with antiviral agents (Guţă et al., 2014), exposing *in vitro* plants to electric fields (Adil et al., 2022), regenerating shoot tips after cryogenic treatment at ultra-low temperatures (Bi et al., 2018), or a combination of these approaches (Mathew et al., 2021; Bettoni et al., 2022), can further increase sanitation success rates. Under optimal culture conditions, it is therefore possible to regenerate virus-free plantlets from excised meristems. Thermotherapy is frequently combined with meristem excision or micrografting to improve sanitation success rates (Wang et al., 2018; Magyar-Tábori et al., 2021; Miljanić et al., 2022). However, while very small meristems (less than 0.5 mm) typically have low regeneration capacity, larger excised meristems (greater than 0.5 mm) can still harbour cells containing intact or degraded virus particles (Maliogka et al., 2009; Szabó et al., 2024).

Chemotherapy offers a promising complementary strategy. Antiviral agents such as ribavirin can intercalate into viral RNA and induce mutations within the replicating viral genome that inhibit further virus accumulation in newly developing plant tissues (Crotty et al., 2002). Plantlets derived from these tissues treated with these compounds can therefore be regenerated virus-free.

Finally, cryotherapy has also been shown effective for eliminating viruses from grapevines (Bettoni et al., 2016; Bi et al., 2018; Marković et al., 2015; Wang et al., 2003). This technique takes advantage of the relative tolerance to freezing of virus-free meristematic cells of the apical dome, which are more likely to survive the ultra-low temperature (-196 °C) of liquid nitrogen, whereas virus-infected older cells perish (Wang and Valkonen, 2009).

This study advances prior virus elimination work in grapevine. First, we report the application of oseltamivir, a neuraminidase inhibitor widely used to treat human influenza (Moscona, 2005), in combination with thermotherapy for GLRaV-3 eradication in multiple grapevine cultivars. Second, we refine existing cryotherapy protocols by incorporating both apical and axillary meristem tips, an approach that may enhance survival and regeneration outcomes. Whereas previous studies, such as those by Bi et al. (2018) and Wang et al. (2003), focused on apical shoot tips, our inclusion of axillary tips adds a second, more abundant source of explant material and thus provides a more efficient system for cryotherapy-based sanitation. Third, our diagnostic strategy combines ELISA, HTS, and Foundation Plant Services Terminal (FPST) RT-qPCR to establish a robust pipeline for comprehensive virus detection. Notably, by targeting the conserved 3′ untranslated region of the GLRaV-3 genome, the FPST RT-qPCR assay offers improved sensitivity and specificity for detecting divergent variants such as NZ2 (Diaz-Lara et al., 2018). Additionally, the inclusion of five distinct grapevine cultivars underscores the critical influence of the host genetic background on the success of tissue regeneration and virus eradication.

We optimised sanitation protocols across several virus-infected grapevine cultivars, establishing a standardised, efficient procedure for germplasm management. The approach could be scaled to restore GLRaV-3-free status, including in mixed-infection accessions and those maintained in geographically isolated repositories, thereby strengthening plant biosecurity and the sustainable management of viticultural resources.

## Materials and methods

### Workflow overview

**Figure 1:**
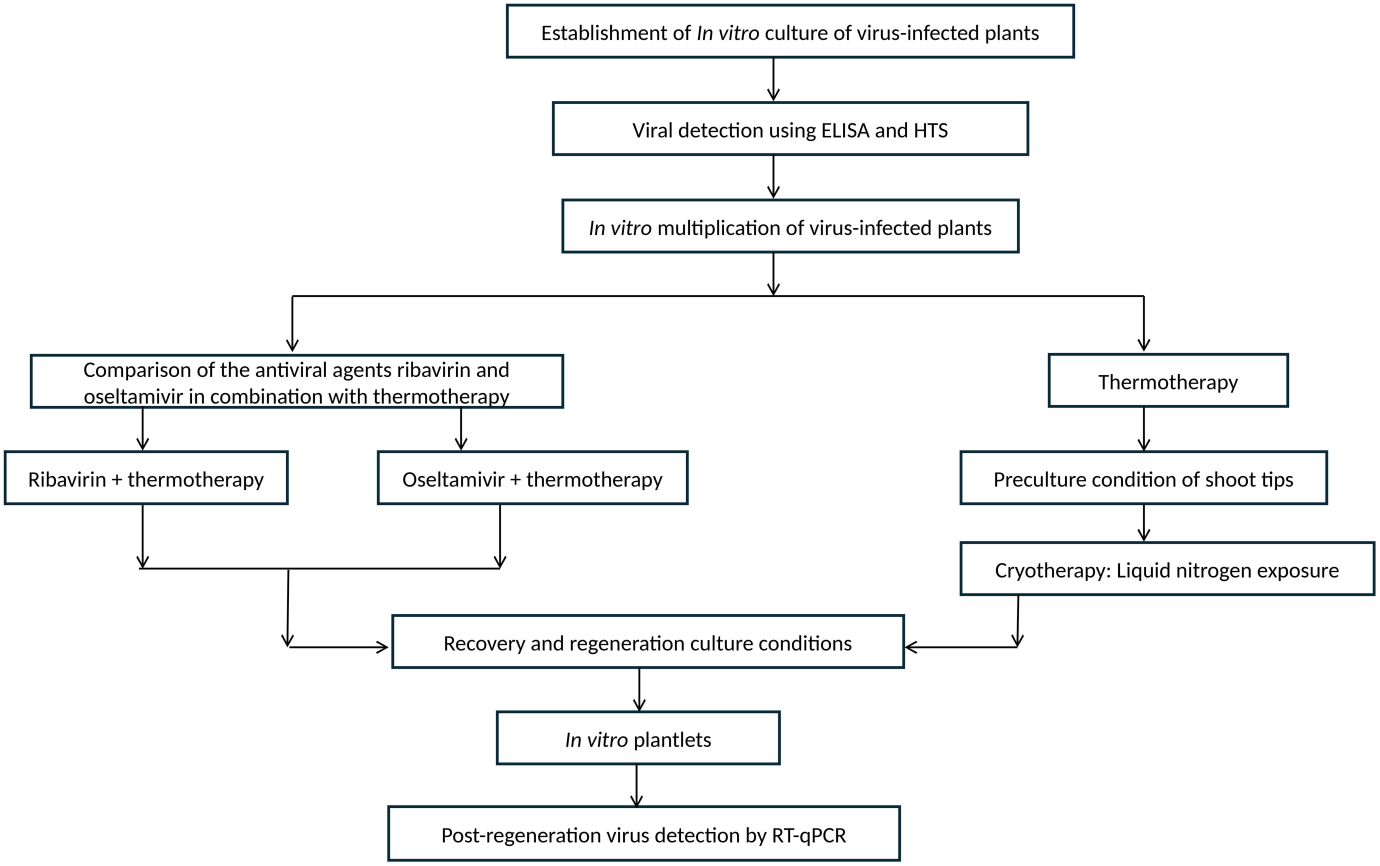
Workflow for GLRaV-3 elimination and post-regeneration detection in *in vitro*–derived plantlets from multiple grapevine cultivars.

Prior to treatment, *in vitro* cultures were established from source plants confirmed to be infected with GLRaV-3 by ELISA and HTS. Cultures were then multiplied *in vitro* and subjected either to thermotherapy combined with the antiviral agents ribavirin or oseltamivir, or to thermotherapy followed by shoot-tip preculture and cryotherapy via liquid-nitrogen exposure (**Figure 1**). Regenerated plantlets were recovered under standard culture conditions and subsequently tested by RT-qPCR to confirm virus elimination.

### Plant material and establishment of aseptic cultures

Fast-growing shoots (approximately 4–6 cm) were collected in October from five grapevine accessions (Chenin Blanc, Ehrenfelser, Riesling Gm 239, Golden Chasselas, and Sauvignon Blanc 217) infected with GLRaV-3 in the low-health section of the NVC (**Table 1**). Opened leaves were removed, and the shoots were rinsed under running tap water to remove surface contaminants. Shoot tips were then surface-sterilised by immersion in 70% ethanol for 30 s, followed by 15–20 min in a solution of 0.5% (w/v) sodium hypochlorite (NaClO) containing two drops of Tween^®^ 20 in 100 mL of solution. Thereafter, the shoots were rinsed 3–5 times with sterile Millipore water. Following surface sterilisation, shoot tips approximately 1.5–2 cm in length were excised and cultured on solidified half-strength Murashige and Skoog (1962) (MS) medium (product number: M0222, Duchefa Biochemie), with 1% (w/v) sucrose and solidified using 0.7% (w/v) Phyto agar. All biochemical reagents used in this study were obtained from Duchefa Biochemie unless otherwise stated. The cultures were maintained in clear, sterile 98 mm (250 mL) tubs (Alto, New Zealand). The medium’s pH was adjusted to 5.8 before autoclaving at 121°C. Shoot tips were plated at a density of five tips per culture tub, and the cultures were maintained in a tissue-culture growth room at 25°C under a 16-h light: 8-h dark photoperiod with a light intensity of 71 µmol m^-^² s^-^¹ provided by cool white daylight fluorescent tubes. After 4 weeks of shoot incubation, the terminal 2 cm of each shoot tip was excised and subcultured to fresh half-strength MS medium for shoot bulking.

**Table 1:** ELISA detection of GLRaV-3 in selected grapevine cultivars from the lower health portion of New Zealand Winegrowers National Vine Collection, as indicated by their respective row and vine positions (vine number) within the row.

No.	Accession no	Cultivar	Row	Vine number	GLRaV-3 status
1	TK05339	Chenin Blanc	10	31	positive
2	TK00031	Ehrenfelser	10	43	positive
3	TK05185	Riesling Gm 239	11	109	positive
4	–	Black Earnscleugh	8	1	negative
5	PN00006	Auxerrois	12	32	negative
6	–	Hermitage Earnscleugh 99157	12	109	negative
7	TK05092	Aligote	9	1	negative
8	TK00066	Gamay Gloroid	10	58	positive
9	–	Golden Chasselas	10	86	positive
10	–	Helena 1	10	94	negative
11	TK06566	Sauvignon Blanc 217	12	6	positive
12	TK00210	Tannat	7	62	positive
13	TK05167	Perle	7	3	positive
14	TK00214	Volta	9	111	positive

### Viral diagnostics

#### Enzyme-linked immunosorbent assay

Mature, fully expanded leaves were collected from mother vines of 14 grapevine genotypes (**Table 1**) located in the low-health section of the New Zealand NVC. Immediately after harvest, the leaves were sealed in clean polyethylene bags, placed on ice, and sent by overnight courier to Hill Laboratories Limited (Blenheim, New Zealand) for testing. Detection of Grapevine leafroll-associated virus 3 (GLRaV-3) was performed using a double antibody sandwich ELISA in a 96-well microtiter plate format. The assay utilised a Bioreba ELISA kit (Bioreba AG, Christoph Merian-Ring 7, CH-4153 Reinach, Switzerland; Lot No. 122373), including coating antibodies (Lot No. 250835), conjugate antibodies (Lot No. 260835), and *p*-nitrophenyl phosphate (pNPP) substrate (Lot No. 282501). Plates were incubated with the coating antibody for 4 hours at 30°C. Following blocking and washing steps according to the manufacturer’s instructions, the plates were incubated with the conjugate antibody for 5 hours at 30°C. Substrate incubation was carried out for 1 hour at 20°C, and absorbance was measured within 5 minutes at a wavelength of 405 nm (OD_405_). Results were interpreted using manufacturer-recommended thresholds. Samples with OD_405_ ≥ 1.500 were classified as positive for GLRaV-3 infection; values <0.100 were classified as negative; values between 0.100 and 1.500 were considered inconclusive and subject to further verification. The positive and negative controls provided with the kit (Lot Nos. 187749 and 101934, respectively) consistently yielded OD values of > 2.000 and < 0.100, within the expected quality control ranges specified in the kit datasheet.

### Validation of diagnostics by HTS

#### RNA extraction for HTS

Mature leaf samples were collected in March from mother vines of the same five grapevine genotypes confirmed to be infected with GLRaV-3 using ELISA. RNA was extracted from approximately 100 mg of frozen mature leaf tissue per sample using the Plant Virus RNA kit (Geneaid Biotech Limited, New Taipei City, Taiwan). The RNA samples were treated using the TURBO DNA-free kit (Invitrogen by Thermo Fisher Scientific Inc., Waltham, MA, United States) to remove genomic DNA contamination. The RNA samples were then quantified using a NanoDrop Eight spectrophotometer (Thermo Fisher Scientific Inc., Waltham, MA, United States) and a Qubit Flex Fluorometer (Thermo Fisher Scientific Inc., Waltham, MA, United States). The integrity of the RNA samples was evaluated using the Bioanalyzer 2100 system with the RNA 6000 Nano kit (Agilent Technologies Inc., Santa Clara, United States).

### RNA sequencing and bioinformatics analysis

Raw sequencing data have been deposited in the National Centre for Biotechnology Information (NCBI) Sequence Read Archive (SRA) under BioProject PRJNA1347987. The RNA samples were processed by Lincoln Genomics at Lincoln University, Canterbury, New Zealand, for ribosomal RNA depletion (QIAGEN QIAseq FastSelect rRNA Plant Kit), cDNA synthesis, and library preparation using the QIAGEN QIAseq UPXome RNA Library Kit for each sample. The libraries were then made MGI-compatible using the MGIEasy Universal Library Conversion Kit (App-A). Sequencing was performed on a DNBSEQ-G400RS sequencer (MGI Tech Co., Ltd, Guangdong, China) using the High-throughput Sequencing Set (FCL PE150).

For analysis, raw sequencing reads were first pre-processed using the nf-core RNAseq pipeline (version v3.17.0-g00f924) to remove adapters, filter low-quality reads, exclude ribosomal RNA and perform quality control checks. Processed reads were then mapped within this pipeline to the grape PN40024 v5 reference genome with the STAR aligner and informed by the PN40024 v5.1 reference annotation. Reads that did not align to the grape reference genome were extracted for further analysis. These unmapped reads were mapped against the NCBI viral reference sequence dataset (parent taxon ‘Viruses’, TaxID 10239), filtered for viruses reported to infect *Vitis vinifera*, downloaded in November 2024. Burrows–Wheeler Aligner-Maximal Exact Match (BWA-MEM, version 0.7.18) was used for this mapping step, as the viral reference sequences were primarily coding sequences, and spliced alignment was not required. Alignments were compressed and indexed using SAMtools (version 1.21), and Mosdepth (version 0.3.4) was used to calculate the mean coverage depth for each viral reference sequence.

To confirm the presence of viruses and viroids, a second approach was employed. Briefly, raw sequencing reads were subjected to quality control, ribosomal RNA removal, and trimming using FastQC (v0.11.7), SortMeRNA (v2.1b) (Kopylova et al., 2012), and Trimmomatic (v0.36) (Bolger et al., 2014), respectively. The processed reads were then aligned to the *Vitis vinifera* cultivar Pinot noir PN40024 v5 reference genome (GCF_030704535.1) using Bowtie2 (v2.3.4.3), and the reads that did not align to the plant genome were extracted. *De novo* assembly of the unaligned reads was performed using SPAdes (v4.0.0) (Prjibelski et al., 2020) and the rnaviralSPAdes pipeline. The resulting contigs were queried against a curated list of viruses and viroids reported from *Vitis* spp. in the NCBI database (as of April 2025), together with unpublished sequences from New Zealand, using blastn (NCBI BLAST v2.11.0).

### RNA extraction and RT-qPCR

**Figure 2:**
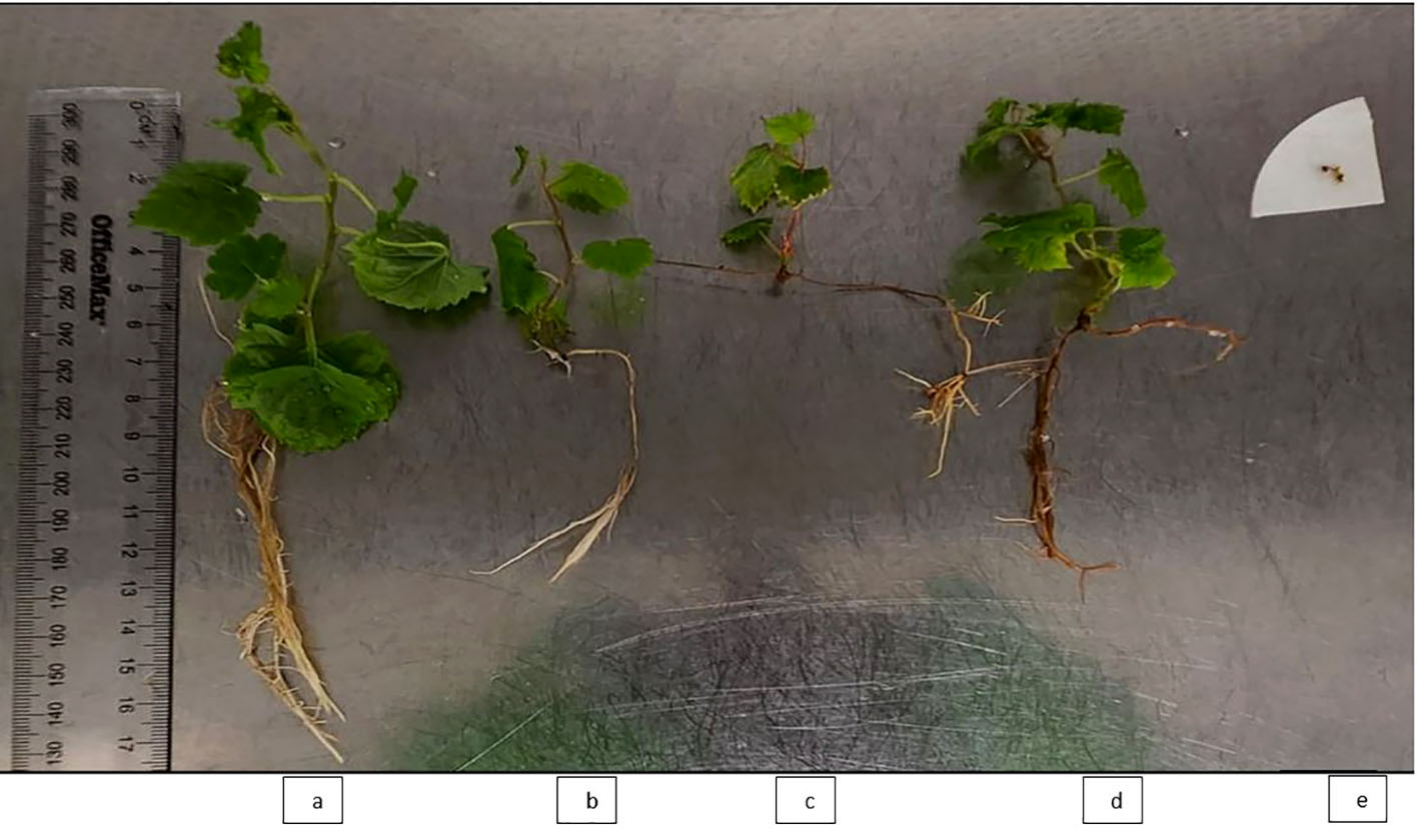
Representative regenerated plantlet grapevine lines used for virus indexing, six weeks post-regeneration, showing developed shoots with roots and leaves: **(a)** Chenin Blanc; **(b)** Ehrenfelser; **(c)** Golden Chasselas; and **(d)** Sauvignon Blanc 217. Panel **(e)** shows a non-viable shoot tip of Riesling Gm 239 (indicated by an arrow) that did not progress to form a plantlet. All plantlets and the shoot tip were obtained following chemotherapy with oseltamivir in combination with thermotherapy.

*In vitro* culture leaf samples were collected from the same five mother grapevine genotypes mentioned above, as well as from the corresponding regenerated plantlet lines derived from virus-elimination treatments (**Figures 2a–d**). Non-viable shoot tips (e.g., **Figure 2e**) were not included in leaf sample collection in this study. Approximately 100 mg of frozen *in vitro* culture leaf samples were ground in liquid nitrogen using a pestle and mortar. RNA was extracted, genomic DNA contamination was removed, and the RNA was quantified as described in Section ‘RNA Extractions for HTS’. To determine the presence or absence of GLRaV-3 in the extracted RNA samples, FPST RT-qPCR was performed using a magnetic induction cycler (Mic) qPCR system (Bio Molecular Systems Pty Ltd, Upper Coomera, Australia) using qScript XLT 1-Step RT-qPCR ToughMix (Quantabio, Beverly, Massachusetts, United States), MGB probes, four forward primers, and two reverse primers as previously described in Diaz-Lara et al., 2018. Each simplex RT-qPCR was performed in a 10 µL reaction containing 2 µL of RNA template. To establish the GLRaV-3 RT-qPCR (FPST) call criteria and limit of detection (LOD), reactions were run in triplicate (unless otherwise specified) using 5 or 10 ng of total RNA per reaction from five pre-treated grapevine cultivars. In addition, RNA from Sauvignon Blanc 217 was tested as an undiluted preparation (159 ng/µL) and as a 10-fold dilution series (8.0, 0.8, and 0.08 ng/µL), each in duplicate. A sample was designated GLRaV-3–positive if a target-specific sigmoidal amplification curve was observed by cycle 40 (quantification cycle (Cq) ≤ 40). Samples with Cq > 40 were classified as below the LOD for GLRaV-3. The same call criteria and LOD were also applied for post-treatment virus testing.

### Comparison of antiviral agents ribavirin and oseltamivir for chemotherapy in combination with thermotherapy

Shoot apices and axillary shoots measuring 0.8 to 1 cm in length were excised from 4-week-old *in vitro* culture grapevine stock material of the five genotypes infected with GLRaV-3. Each genotype was represented by twenty shoots, arranged as five shoots per culture tub. The cultures were incubated for two weeks on fresh solidified half-strength MS medium as described above. Following this initial incubation, explants were transferred to fresh half-strength MS medium supplemented with either 25 μg mL^-^¹ ribavirin (Hu et al., 2020) or 100 μmol L^-^¹ oseltamivir phosphate (both from Thermo Scientific, USA) and 1% (w/v) sucrose. The medium was solidified with 0.7% (w/v) Phyto agar. For the oseltamivir treatment, the culture medium was supplemented with 10 μM salicylic acid (SA). Given the limited reports on the use of oseltamivir for plant virus elimination, SA was included to potentially mitigate phytotoxic effects (Pathirana et al., 2016). This differs slightly from Hu et al. (2020), who used oseltamivir without SA for virus elimination. The cultures were subjected to a stepwise temperature regime, starting at 25°C for the first week, then 30°C in the second week, and 35°C for the third and fourth weeks, again differing from Hu et al. (2020), who used a constant temperature throughout the treatment. The plant cultures were maintained in a tissue culture growth cabinet providing 44 µmol m^-^² s^-^¹ of light intensity, with a 16-h photoperiod.

At the end of the 4-week treatment, shoot tips measuring 0.8 to 1 mm in length and containing two to three leaf primordia were excised from grapevines treated with either ribavirin or oseltamivir. These shoot tips were cultured on half-strength basal MS medium (product number: M0221, Duchefa Biochemie). Initially, the shoot tip cultures were incubated in 35 mm disposable sterile plastic Petri dishes and maintained in the dark at 25°C for 2 weeks. After this 2-week dark incubation, shoots were transferred to culture tubs containing fresh half-strength MS medium as described under culture establishment. The cultures were maintained in a growth room under the same conditions as stock cultures. They were subcultured every fourth week for 8–10 weeks to develop into plantlets, which were subsequently tested for GLRaV-3 by RT-qPCR.

### Thermotherapy followed by cryotherapy

#### *In vitro* thermotherapy

Shoot apices and axillary shoots measuring 0.8 to 1 cm in length were excised from tissue-culture grapevine stock material of the five genotypes infected with GLRaV-3. For each genotype, six tissue-culture tubs, each containing five shoots, were established. These cultures were incubated for 2 weeks on fresh solidified half-strength MS medium as described for the stock cultures. The tissue culture growth cabinet was initially set at 25°C for 2 weeks, then programmed to increase by 5°C during the third and fourth weeks. Subsequently, the temperature was maintained at 35°C for the fifth week. A 16 h photoperiod was used throughout, with a photon-flux density of 44 µmol m^-^² s^-^¹. Extending thermotherapy to five or six weeks significantly increased the proportion of virus-free plants when no chemotherapy was applied and the shoot tips were subsequently subjected to cryotherapy (Zhao et al., 2018).

### Pretreatment and cryotherapy

#### Preculture condition

Following thermotherapy, apical shoot tips measuring ≤ 1 mm in length, with two to three leaf primordia, were excised from fifteen randomly selected shoots of each genotype and cultured in the preculture medium on sterile filter paper for 3 days in the dark. Similarly, from the remaining fifteen shoots of each genotype, both apical shoot tips and axillary bud tips measuring ≤ 1 mm in length were excised and cultured in the fresh preculture medium on sterile filter paper for 3 days in the dark. The preculture medium was composed of half-strength MS medium (Product number. M0222, Duchefa Biochemie), supplemented with 0.3 M sucrose, 0.1 mM SA, 0.25 mM ascorbic acid, and 0.25 mM reduced glutathione, solidified using 0.7% (w/v) Phyto agar, with pH adjusted to 5.8 before autoclaving, following a modified protocol based on Bettoni et al. (2020). Cultures were maintained in darkness at 25°C for 3 days. After 3 days of dark incubation, the cultures of apical shoot tips and the combined apical shoot and axillary bud tips were transferred to a loading solution for 20 min. The loading solution comprised half-strength MS medium (product number: M0222, Duchefa Biochemie), 0.4 M sucrose, and 2 M glycerol, with the pH adjusted to 5.8 before autoclaving, following the method described by Bettoni et al. (2020).

Subsequently, the apical shoot tips and the combined apical shoot and axillary bud tips were transferred to half-strength Plant Vitrification Solution 2 (PVS2) under aseptic conditions in a laminar flow hood for 30 min at room temperature. The half-strength PVS2 consisted of half-strength MS medium (product number: M0222, Duchefa Biochemie), 15% (w/v) glycerol, 7.5% (w/v) ethylene glycol, 7.5% (w/v) dimethyl sulfoxide, and 0.4 M sucrose, with the pH adjusted to 5.8 and prepared as a filter-sterilised solution (modified from Sakai et al., 1990). Afterwards, the shoot tips underwent 60 min of incubation under aseptic conditions in a laminar flow hood at 0°C in full-strength PVS2. The full-strength PVS2 included full-strength MS medium (product number: M0222, Duchefa Biochemie), 30% (w/v) glycerol, 15% (w/v) ethylene glycol, 15% (w/v) dimethyl sulfoxide, and 0.4 M sucrose, with the pH adjusted to 5.8, prepared as a filter-sterilised solution (Bettoni et al., 2020).

### Liquid nitrogen exposure

At the end of incubation in PVS2 solution, the apical shoot tips and the combined apical shoot and axillary bud tips were immediately placed onto sterilised foil strips coated with a thin layer of PVS2. They were then briefly immersed in liquid nitrogen before being quickly transferred into 2-mL cryovials (Greiner Bio-One, Frickenhausen, Germany) already submerged in liquid nitrogen. The samples were left in liquid nitrogen for 60 min, following the method described by Bettoni et al. (2020). After 60 min of liquid nitrogen exposure, the foil strips containing the apical shoot tips and the combined apical shoot and axillary bud tips were removed from the liquid nitrogen and promptly transferred to an unloading solution under aseptic conditions in a laminar flow hood and held at room temperature for 20 min. The unloading solution consisted of filter sterilised half-strength MS medium (product number: M0222, Duchefa Biochemie) and 1.2 M sucrose, adjusted to pH 5.8.

### Recovery and regeneration culture conditions

Following the thawing of the apical shoot tips and the combined apical shoot and axillary bud tips in the unloading solution, they were cultured on recovery medium #1 (Bettoni et al., 2020). This recovery medium consisted of a mixture of half-strength MS basal salts (product number: M0221, Duchefa Biochemie), half-strength MS microelements (product number: M0301, Duchefa Biochemie), Vitis vitamins, supplemented with 0.6 M sucrose, and solidified with 0.7% (w/v) Phyto agar, adjusted to pH 5.8 before autoclaving. The apical shoot tips and the combined apical shoot and axillary bud tips were incubated on sterilised filter paper placed on recovery medium #1 in 90 mm disposable sterile plastic Petri dishes and maintained overnight in the dark at 25°C.

After the overnight incubation in recovery medium #1, the apical shoot tips and the combined apical shoot and axillary bud tips were transferred to recovery medium #2 for 2 weeks and kept in the dark. This medium consisted of half-strength MS basal salts (product number: M0221, Duchefa Biochemie), half-strength MS microelements (product number: M0301, Duchefa Biochemie), Vitis vitamins, 0.1 M sucrose, 0.2 mg/L 6-benzylaminopurine (BAP), and was solidified with 0.7% (w/v) Phyto agar. The pH was adjusted to 5.8 before autoclaving. After 2 weeks of incubation, the apical shoot tips and the combined apical and axillary bud tips were transferred to a fresh recovery medium #2 for 8 weeks to promote shoot elongation, achieving lengths of 1–2 cm. During the first week, the cultures were exposed to a tissue-culture growth chamber light intensity of 22 µmol m^-^² s^-^¹ at 25 °C, then increased to 44 µmol m^-^² s^-^¹ for the remaining weeks, under a 16-h photoperiod. This experimental setup aimed to assess survival and regrowth. Shoot regrowth that reached 1–2 cm was transferred to fresh, solidified half-strength MS medium (product number: M0222, Duchefa Biochemie), supplemented with 1% (w/v) sucrose and solidified with 0.7% (w/v) Phyto agar. The cultures were maintained in a growth room at 25°C under a 16-h photoperiod with a light intensity of 71 µmol m^-^² s^-^¹, provided by cool white daylight fluorescent tubes. They were subcultured every fourth week for 8–10 weeks to develop into plantlets, which were subsequently evaluated.

### Data analysis

For each cultivar–treatment combination, we used three to four independent culture tubs (biological replicates), each containing five plantlets (subsamples). Treatments were randomised to tubs. Statistical analyses were performed in RStudio (version 2023.12.1 + 402). We defined three endpoints: (i) survival at 4 weeks (surviving shoot tips/excised tips), (ii) regeneration conditional on survival (regenerated independent shoots/surviving tips), and (iii) number of plantlet lines per cultivar. For each group, we calculated the sample propor-on (p) and plored the es-mate ± binomial standard error. Pairwise group comparisons were performed using Fisher’s exact tests, with p-values adjusted for mul-ple tes-ng using the Benjamini–Hochberg procedure (α = 0.05). Significant groupings were summarised using compact lerer displays; groups sharing a lerer were not significantly different under the BH-adjusted Fisher approach.

For the HTS virus analysis, results were analysed using RStudio Pro running R version 4.3.3 (R Core Team, 2024). For each detected virus and viroid, reads were mapped to the reference genome with the highest nucleotide identity, and genome coverage and read counts were generated.

## Results

### Detection of virus infections in the New Zealand NVC held in Lincoln

ELISA was used to detect GLRaV-3 in selected grapevine cultivars from the low-health section of the NVC. Nine out of fourteen tested cultivars (64.3%) were GLRaV-3 positive (**Table 1**), confirming a high prevalence of infection within the collection. In contrast, five cultivars (35.7%) tested negative for GLRaV-3 by ELISA (**Table 1**). HTS of the mother vines was conducted. **Tables 2** and **3** present the results of high-throughput RNA sequencing used to detect viruses and viroids in selected mother grapevine cultivars. Viroids such as grapevine yellow speckle viroid 1 were detected exclusively in Sauvignon Blanc 217, whereas hop stunt viroid was identified across five different cultivars (**Table 2**). The sequencing analysis identified mixed grapevine viral infections across five cultivars: Sauvignon Blanc 217, Ehrenfelser, Chenin Blanc, Riesling Gm 239, and Golden Chasselas, as shown in **Table 3**. Among the detected viruses, a member of the genera Vitivirus and Ampelovirus were prevalent, with grapevine virus G (GVG) and GLRaV-3 detected in all cultivars. Ehrenfelser exhibited the highest number of detected viruses, whereas Riesling Gm 239 and Golden Chasselas showed the lowest. Other grapevine viruses identified included the relatively recently described grapevine red globe virus (GRGV) and grapevine rupestris vein feathering virus (GRVFV), which belong to the genera Maculavirus and Marafivirus, respectively. The GRGV was detected in Sauvignon Blanc 217, whereas GRVFV was present in Chenin Blanc and Golden Chasselas. These findings, presented in **Table 3**, confirm the presence of a diverse range of mix grapevine virus infections across the selected cultivars, highlighting the significance of optimising treatment strategies for effective virus elimination within the NVC.

**Table 2:** Summary of libraries, corresponding grapevine cultivars, paired-end sequencing reads, number of viruses identified, and detected viroids.

Library	Cultivar	Paired-end sequencing reads	No. of detected viruses	Detected viroid
G10	Sauvignon Blanc 217	9,836,542	5	Grapevine yellow speckle viroid 1 and Hop stunt viroid
A10	Ehrenfelser	5,026,721	6	Hop stunt viroid
C10	Chenin Blanc	2,909,744	5	Hop stunt viroid
D10	Riesling Gm 239	6,779,359	3	Hop stunt viroid
E10	Golden Chasselas	1,568,389	3	Hop stunt viroid

**Table 3:** High-throughput RNA sequencing results confirming the detection of grapevine viruses in selected mother grapevine cultivars.

Cultivar	Virus detected^1^	Reference genbank accession No.	Reference genome size (nucleotides)	Genome coverage (%)	Read count
Sauvignon Blanc 217	Grapevine leafroll-associated virus 2	OR640977	16427	100	337855
	Grapevine leafroll-associated virus 3	ON868738	18624	93.7	597662
	Grapevine Red Globe virus	NC_030693	6863	94.1	1485
	Grapevine rupestris stem pitting-associated virus	LC746723	8711	98.6	27100
	Grapevine virus G	NC_040616	7496	100	499300
Ehrenfelser	Grapevine asteroid mosaic associated virus	NC_031692	6719	98.3	3083
	Grapevine leafroll-associated virus 3	ON868738	18624	99.4	63517
	Grapevine Syrah virus 1	NC_012484	6506	98.5	1691
	Grapevine virus A	OP752638	7380	62.8	9416
	Grapevine virus G	NC_040616	7496	100	41531
	Grapevine virus I	NC_037058	7507	100	19103
Chenin Blanc	Grapevine leafroll-associated virus 3	ON868739	18476	97.3	49088
	Grapevine rupestris vein feathering virus	NC_034205	6730	44.1	225
	Grapevine virus A	OP752608	7183	98.9	8799
	Grapevine virus G	NC_040616	7496	100	34900
	Grapevine virus I	NC_037058	7507	100	83213
Riesling Gm 239	Grapevine leafroll-associated virus 3	JX220900	18538*	11.9	15294
	Grapevine rupestris stem pitting-associated virus	ON868747	8758	79.1	8988
	Grapevine virus G	NC_040616	7496	100	261331
Golden Chasselas	Grapevine leafroll-associated virus 3	JX220899	18617*	17.8	34514
	Grapevine rupestris vein feathering virus	NC_034205	6730	77.9	658
	Grapevine virus G	NC_040616	7496	100	55082

^(a)^Grey shading indicates virus detections with low read counts or limited genome coverage relative to the reference genome.

^(b)^Includes sequence data not currently available on the National Centre for Biotechnology Information.

### Plant survival and regeneration after *in vitro* therapies for virus elimination

#### Plant survival and regeneration after thermotherapy combined with ribavirin-based chemotherapy

**Figure 3:**
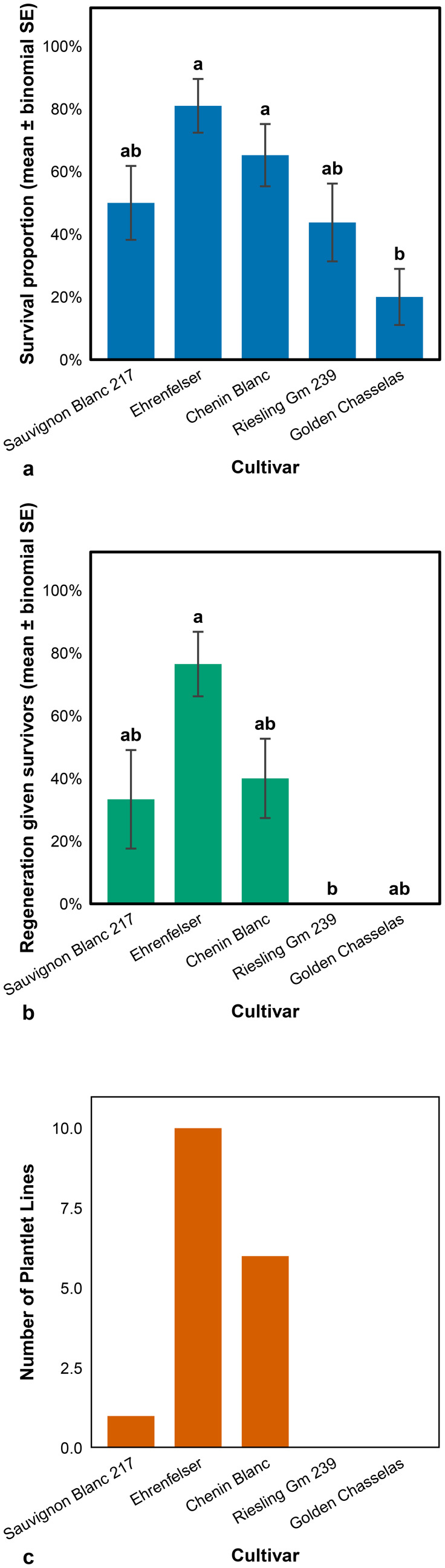
Survival and plantlet regeneration (shoots with developed roots and leaves) of grapevine apical microshoots following combined *in vitro* chemotherapy (ribavirin) and thermotherapy. **(a)** Shoot-tip survival at 4 weeks (estimate ± binomial standard error.), **(b)** Independent shoots regenerated (among survivors) (mean ± SEM), and **(c)** Number of plantlet lines by cultivar.

**Figures 3a, b** present survival and regeneration outcomes after a combined *in vitro* chemotherapy treatment with ribavirin and thermotherapy. Despite the same starting explant material, the number of apical shoot tips excised varied slightly among cultivars. Chenin Blanc and Ehrenfelser had the highest mean number of tips (22), whereas the remaining cultivars averaged 18. The survival rate after 4 weeks was highly variable across cultivars, ranging from 20% surviving shoots in Golden Chasselas to 81% in Ehrenfelser, indicating cultivar-dependent responses to the treatment, as shown in **Figure 3a**. In this study, apical shoot tip survival was defined as the transition in shoot tip colouration from light green or brownish to green, observed 4 weeks following treatment. The ability of surviving shoots to regenerate independent shoot lines also varied among cultivars, as shown in **Figure 3b**. Ehrenfelser exhibited the highest regeneration rate, followed by Chenin Blanc and Sauvignon Blanc 217, while Riesling Gm 239 and Golden Chasselas did not regenerate independent shoot lines. Regenerated independent shoot lines further developed into plantlets, characterised by the differentiation of shoots into leaves, stems, and roots. The highest number of plantlets available for viral screening was in Ehrenfelser, followed by Chenin Blanc and Sauvignon Blanc 217 (**Figure 3c**). In contrast, Riesling Gm 239 and Golden Chasselas had no plantlets available for testing, as shown in **Figure 3c**, indicating their limited survival and regeneration potential under these treatment conditions.

#### Plant survival and regeneration after thermotherapy combined with oseltamivir-based chemotherapy

**Figure 4:**
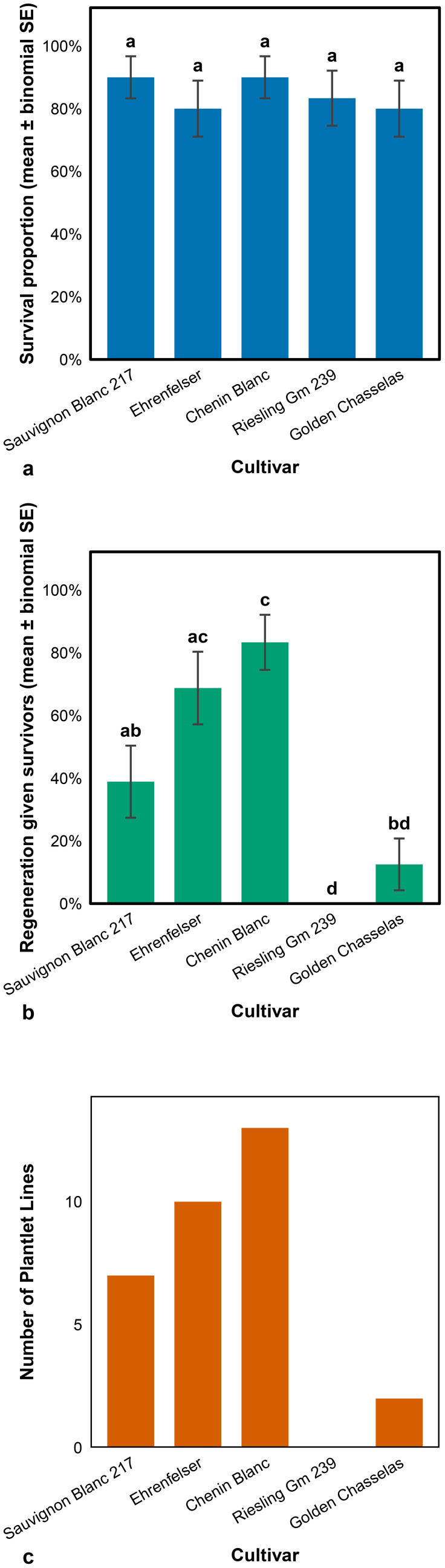
Survival and plantlet regeneration (shoots with developed roots and leaves) of grapevine apical microshoots following combined *in vitro* chemotherapy (oseltamivir) and thermotherapy. **(a)** Shoot-tip survival at 4 weeks (estimate ± binomial standard error.), **(b)** Independent shoots regenerated (among survivors) (mean ± SEM), and **(c)** Number of plantlet lines by cultivar.

**Figure 4** shows the survival and regeneration outcomes of apical microshoots from the five grapevine cultivars following a combined *in vitro* chemotherapy treatment with oseltamivir and thermotherapy. Twenty apical microshoot tips were excised from each cultivar; Riesling Gm 239 had the fewest, with 18. However, in Riesling Gm 239, this was affected by the failure of other shoots to develop into plantlets capable of producing apical tips. Apical shoot tip survival was defined as the transition in shoot tip colouration from light green or brownish to green, observed 4 weeks following treatment (**Figure 4a**). Significant variation was observed across cultivars with regards to regeneration potential, with Riesling Gm 239 failing to generate any independent shoot lines (**Figure 4b**). Sauvignon Blanc 217 and Chenin Blanc exhibited the highest shoot tip survival rate. Most Chenin Blanc regenerated shoot lines developed further into plantlets, as shown in **Figure 4c**, and were characterised by the differentiation of shoots into leaves, stems, and roots, as illustrated in **Figure 2a**.

### Explant survival and plant regeneration: thermotherapy followed by cryotherapy

**Figure 5:**
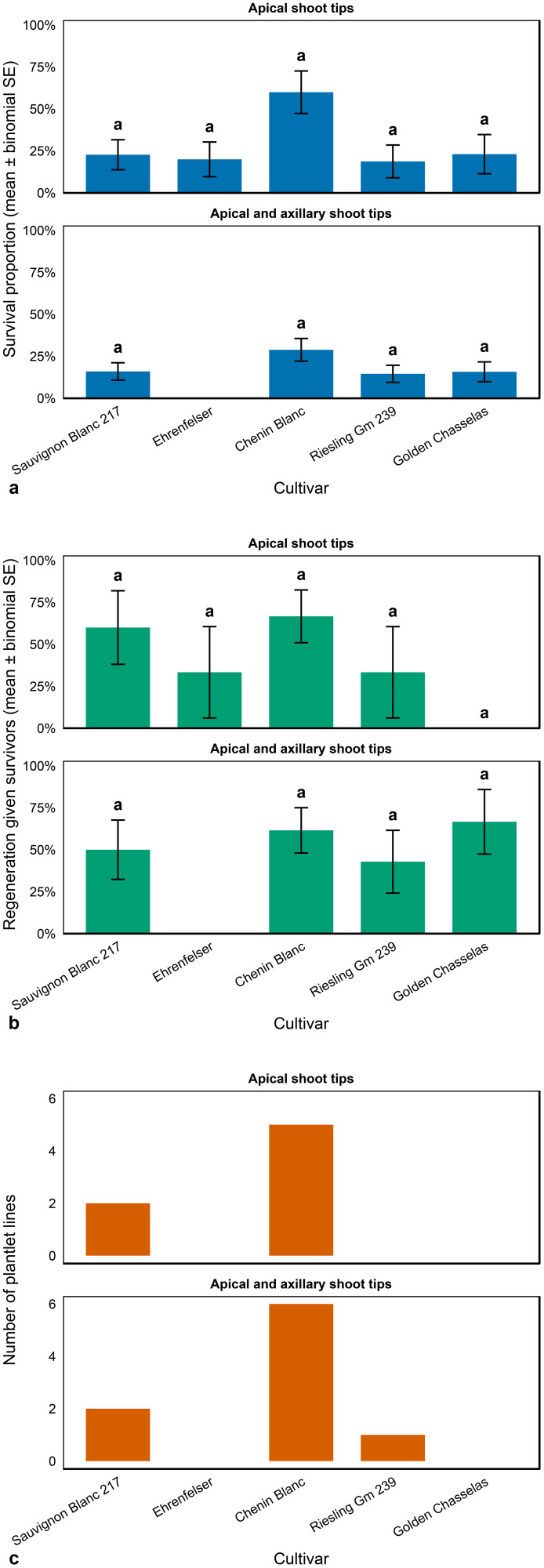
Survival and plantlet regeneration (shoots with developed roots and leaves) of grapevine apical microshoot tips and combined apical and axillary shoot tips following *in vitro* thermotherapy, followed by cryotherapy. **(a)** Shoot-tip survival at 4 weeks (estimate ± binomial standard error.), **(b)** Independent shoots regenerated (among survivors) (mean ± SEM), and **(c)** Number of plantlet lines by cultivar. Owing to technical difficulties, combined apical and axillary shoot tips could not be excised from Ehrenfelser plants, precluding a direct comparison of shoot survival among treatments.

**Figure 6:**
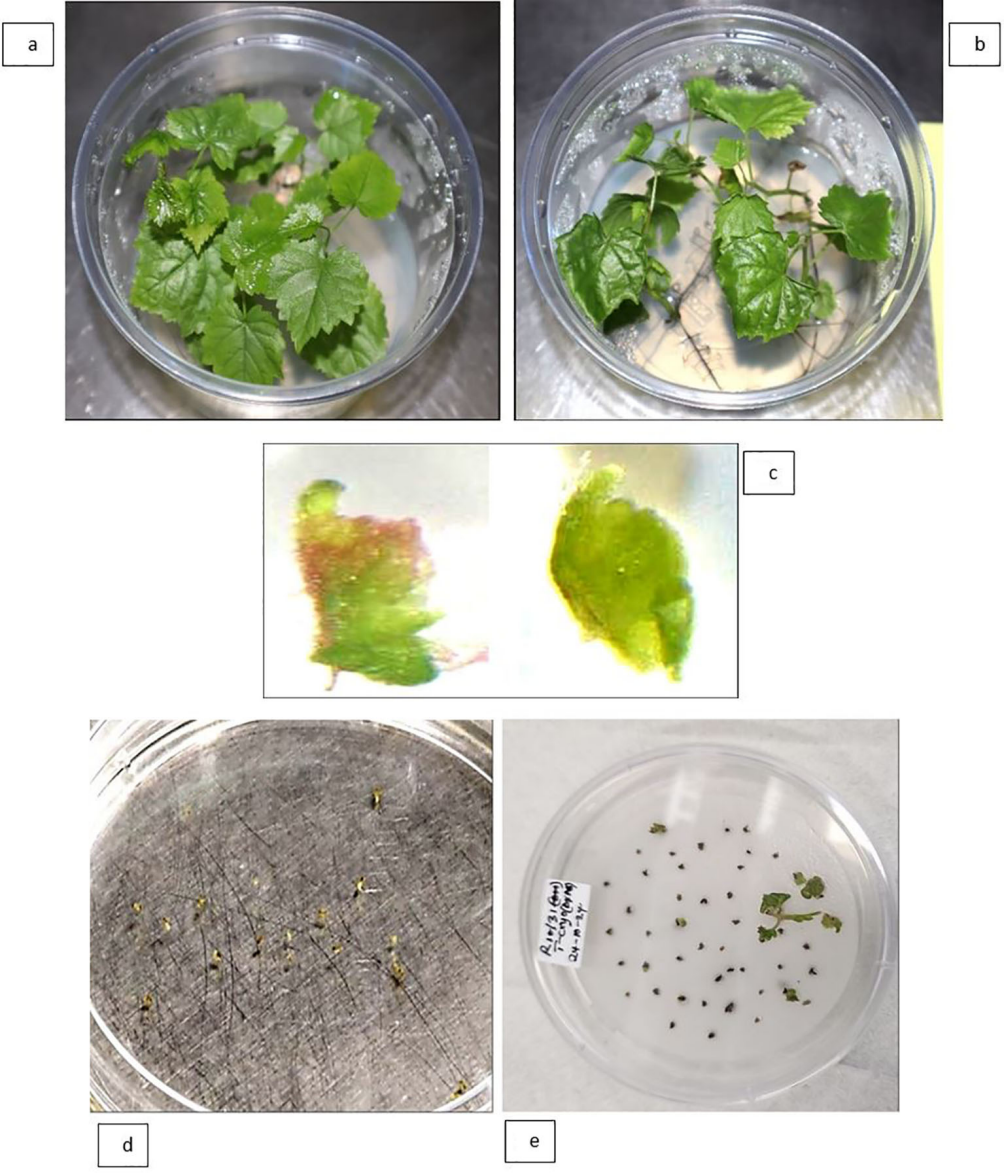
Demonstration of stress response in grapevine plantlets and shoot tips and plant regeneration of Grapevine leafroll-associated virus-3-infected Chenin Blanc during thermotherapy followed by cryotherapy. **(a)** Control plantlets growing at an average temperature of 25 °C. **(b)** Plantlets subjected to thermotherapy at 35 °C prior to shoot tip excision. **(c)** Representative excised shoot tips (≤1 mm long, 100× magnification) containing two to three leaf primordia were prepared for cryotreatment. **(d)** Shoot tips recovered after 1 hour of immersion in liquid nitrogen. **(e)** Cryotreated shoot tips on recovery medium #2. Most cells were injured; however, surviving meristematic cells regenerated into shoots 4 weeks after cryotreatment.

The results presented in **Figures 5a,b** provide insights into the survival and regeneration capacity of apical micro shoot and axillary shoot tips from various grapevine cultivars following *in vitro* thermotherapy combined with cryotherapy, while the corresponding treatment effects are illustrated in **Figure 6**. The treatment protocol involved subjecting GLRaV-3-infected grapevine plantlets to thermotherapy at 35°C, followed by excision of shoot tips and cryopreservation in liquid nitrogen. Regeneration was subsequently carried out on Recovery Media #1 and #2.

Among the tested cultivars, Chenin Blanc exhibited the highest regenerative response, producing five independent plantlet lines from apical shoot tips, as shown in **Figure 5c**, which met the criteria suitable for GLRaV-3 virus indexing in this study (a shoot with two or more developed roots and three or more leaves). Inclusion of both apical and axillary shoot tips further enhanced regeneration, yielding six independent plantlet lines, suggesting that the incorporation of axillary tips increases the overall regeneration efficiency.

In Riesling Gm 239, the combined use of apical and axillary tips also improved regeneration outcomes, resulting in the development of one virus-indexable plantlet line (**Figure 5c**). Overall, the data indicate that the use of both apical and axillary shoot tips generally enhances survival rates and the number of regenerated, independent shoot lines across most cultivars.

**Figure 6** illustrates the procedural workflow and associated stress responses of GLRaV-3–infected grapevine plantlets and shoot tips during cryotreatment and subsequent regeneration. Untreated *in vitro* plantlets (**Figure 6a**) served as controls and exhibited normal vegetative growth, whereas heat-preconditioned plantlets (**Figure 6b**) showed pronounced heat stress symptoms, including reduced vegetative growth. From these preconditioned plants, representative shoot tips (≤1 mm in length; 100× magnification), each containing two to three leaf primordia, were excised and prepared for cryotreatment (**Figure 6c**). After 1 h of immersion in liquid nitrogen, most shoot tips survived the cryogenic exposure (**Figure 6d**). During the recovery phase, although many cells were damaged, viable meristematic cells persisted and successfully regenerated into shoots (**Figure 6e**).

### Comparative GLaV-3 elimination efficiency by FPST RT-qPCR

Baseline (pre-treatment) FPST RT-qPCR assays w**i**th 5 ng total RNA per reaction yielded mean Cq (± SD) values ranging from 17.57 ± 0.05 to 21.61 ± 0.72 across the five cultivars (**Table 4**). Doubling the template RNA input to 10 ng per reaction produced the expected modest Cq decrease (≈0.2–0.9 cycles) as shown in **Table 4**. Baseline amplification plots and dilution-series analytical sensitivity are shown in **Supplementary Figure S1**. FPST RT-qPCR was further evaluated using pre-treated Sauvignon Blanc 217 RNA at 159 ng/µL and a 10-fold dilution series (8.0, 0.8, 0.08 ng/µL), each tested in duplicate; amplification was still observed at the lowest concentration (0.08 ng/µL), confirming GLRaV-3 detection (**Supplementary Figure S2**).

**Table 4:** FPST RT-qPCR performance for GLRaV-3 across five grapevine cultivars at 10 and 5 ng/µL RNA input, showing mean Cq ± SD, amplification efficiency, and linearity (R²).

Cultivar	RNA 5ng/ul			RNA 10ng/ul		
	Mean Cq value (± SD)	Efficiency	R^2^	Mean Cq value (± SD)	Efficiency	R^2^
Sauvignon Blanc 217	17.95 ± 0.65	0.92	0.999	17.15 ± 0.14	0.98	0.998
Ehrenfelser	18.61 ± 0.04	0.93	0.999	18.41 ± 0.39	0.94	0.998
Chenin Blanc	19.08 ± 0.14	0.95	0.999	18.37 ± 0.14	0.99	0.999
Riesling Gm 239	21.61 ± 0.72	0.87	0.998	20.71 ± 0.10	0.87	0.998
Golden Chasselas	17.57 ± 0.05	0.92	0.999	17.42 ± 0.08	0.94	0.998

Across treated lines of Sauvignon Blanc 217, Ehrenfelser, and Riesling Gm 239, neither thermotherapy plus cryotherapy nor thermotherapy–chemotherapy with oseltamivir yielded detectable GLRaV-3 by RT-qPCR through 40 cycles. No-template and negative controls were likewise non-amplifying, supporting assay specificity. With ≥10 ng total RNA per reaction, all treated samples were therefore classified as below the limit of detection (LOD) (**Supplementary Figure S3**).

The effectiveness of *in vitro* virus-elimination treatments across all five cultivars is summarised in **Table 5**. Treatments included chemotherapy with ribavirin or oseltamivir combined with thermotherapy, and thermotherapy followed by cryotherapy. Virus status of regenerated plants was assessed by RT-qPCR using leaves collected 6 or 8 weeks after regeneration. The FPST RT-qPCR performed robustly across all cultivars and RNA inputs. As expected for a two-fold input increase, 10 ng per reaction produced modestly lower Cq values than 5 ng (≈0.2–0.9 cycles), indicating a proportional response. Amplification efficiencies were high (92–99%) with excellent linearity (R² ≥ 0.998) and low within-run variability (SD ≤ 0.72; typically, ≤ 0.39). Riesling Gm 239 consistently showed higher Cq and slightly reduced efficiency (87%) at both inputs, suggesting lower template abundance and/or minor matrix inhibition. Overall, GLRaV-3 was reliably detected in the pre-treated grapevine cultivars across inputs.

**Table 5:** Diagnostic summary of grapevine leafroll-associated virus-3 elimination efficiency using qRT-PCR on grapevine plantlet lines subjected to combined *in vitro* chemotherapy (either ribavirin or oseltamivir) and thermotherapy, and *in vitro* thermotherapy followed by cryotherapy.

Cultivar	Explant types	GLRaV-3 free lines among the regenerants % (n)		
		Combined *in vitro* chemotherapy (ribavirin) and thermotherapy	Combined *in vitro* chemotherapy (oseltamivir) and thermotherapy	*In vitro* thermotherapy and cryotherapy
Sauvignon Blanc 217	AST	0 (0/1)	14.3 (1/7)	100 (2/2)
	AXTs	–	–	100 (2/2)
Ehrenfelser	AST	20 (2/10)	100 (10/10)	0
	AXTs	–	–	-
Chenin Blanc	AST	33.3 (2/6)	38. 5 (5/13)	100 (5/5)
	AXTs	–	–	100 (6/6)
Riesling Gm 239	AST	0	0	0
	AXTs	–	–	100 (1/1)
Golden Chasselas	AST	0	100 (2/2)	0
	AXTs	–	–	0

^(a)^AST, apical shoot tip; AXTs, apical and axillary tips. Consequently, GLRaV-3 elimination efficiency could not be compared using qRT-PCR with those of plantlet lines derived from apical shoot tips or the apical and axillary tips of other cultivars.

### Ribavirin chemotherapy combined with thermotherapy

The efficiency of chemotherapy with ribavirin when combined with thermotherapy varied among the five cultivars. Sauvignon Blanc 217 did not yield any virus-free plantlets (0%). In contrast, Ehrenfelser exhibited partial virus elimination, with 20% of plantlets testing negative. Chenin Blanc demonstrated a virus elimination rate of 33.3%. However, Riesling Gm 239 and Golden Chasselas failed to regenerate viable plantlets, precluding the determination of the percentage of GLRaV-3-free plantlets.

### Oseltamivir chemotherapy combined with thermotherapy

Compared with ribavirin, chemotherapy with oseltamivir, when combined with thermotherapy, exhibited moderate to high virus elimination rates. Sauvignon Blanc 217 achieved a 14.3% virus-free plantlet rate, whereas Ehrenfelser showed complete virus elimination (100%). Chenin Blanc recorded a 38.5% elimination rate. Similarly to the previous treatment, Riesling Gm 239 did not regenerate any plantlets, preventing the determination of its GLRaV-3 elimination rate. Conversely, Golden Chasselas achieved complete virus elimination, with 100% of plantlets testing negative.

### Thermotherapy followed by cryotherapy

Thermotherapy, when followed by cryotherapy, yielded the highest elimination rates among all treatments. Sauvignon Blanc 217 achieved complete elimination (100%). Owing to technical challenges encountered with apical and axillary tips, Ehrenfelser was not evaluated for this treatment. Chenin Blanc exhibited 100% virus elimination in both apical shoot tips and apical and axillary tips. Riesling Gm 239 also achieved complete elimination (100%) in apical and axillary tips; however, no viable plantlets were generated from apical shoot tips, preventing the assessment of its GLRaV-3 status.

## Discussion

Grapevine leafroll-associated virus 3 (GLRaV-3) was detected by ELISA in most cultivars that were randomly-selected from the low-health section of the NVC. HTS RNA sequencing analysis of these mother grapevine cultivars revealed a diverse virome, including well-characterised grapevine viruses, recently reported viral species such as grapevine red globe virus (GRGV) and grapevine rupestris vein feathering virus (GRVFV) (Candresse et al., 2023), and viroids. In addition to the confirmed presence of members of the genus Ampelovirus (e.g., GLRaV-3), which were widely detected across all cultivars, vitiviruses such as grapevine virus G (GVG) were also consistently present across the cultivars. Furthermore, a member of the *Closteroviridae* family, grapevine leafroll-associated virus 2 (GLRaV-2), which has been reported to be associated with Grapevine Leafroll Disease (GLD), was detected only in accession 217. This is consistent with the findings of Sharma et al. (2011), who reported that only approximately 15% of 134 GLD-infected grapevines in Napa Valley (California, USA) vineyards had mixed infections of GLRaV-3 with GLRaV-1, -2, -4, or -9. Our findings, along with the study by Xiao and Meng (2023) in Ontario, Canada, confirm the presence of a diverse viral landscape in the selected grapevine cultivars and reinforce the need for optimised virus elimination strategies in the NVC and other grapevine germplasm collections infected with similar viruses.

We report here the comparative effectiveness of virus elimination strategies for GLRaV-3 across multiple grapevine cultivars from the NVC. In this study, baseline Cq values (17.6–21.6 at 5 ng RNA) and the predictable ≈0.2–0.9-cycle decrease at 10 ng indicate proportional assay response to input, consistent with expected qPCR behaviour and Minimum Information for qPCR experiments (MIQE) guidance for performance reporting (Bustin et al., 2009). The Sauvignon Blanc 217 dilution series, which remained detectable down to 0.08 ng/µL, supports good analytical sensitivity; comparable GLRaV-3 RT-qPCR performance (including the FPST assay) has been reported for diverse variants (Diaz-Lara et al., 2018). Assay quality metrics here (efficiency 92%–99%; R² ≥ 0.998; low within-run standard deviations) meet widely accepted benchmarks, indicating that observed differences predominantly reflect biological rather than technical variance (Bustin et al., 2009). Importantly, no-template controls (NTCs) and negative controls remained negative with no amplification or melt curve peaks through 40 cycles in all runs, satisfying MIQE-recommended contamination safeguards and minimising concern that positives arose from carryover or primer-dimer artefacts (Bustin et al., 2009).

Post-treatment, the lack of amplification by cycle 40 in Sauvignon Blanc 217, Ehrenfelser, and Riesling Gm 239, even when RNA input was ≥ 10 ng per reaction, indicates that thermotherapy plus cryotherapy, or thermotherapy combined with chemotherapy, reduced GLRaV-3 to below the limit of detection, consistent with previous sanitation studies in grapevine (Bi et al., 2018; Bettoni et al., 2021; Hu et al., 2020). The consistently higher Cq values and slightly lower efficiency in Riesling Gm 239 are compatible with a lower initial viral load and/or mild matrix inhibition, a known issue in plant qPCR that varies by tissue and cultivar (Schrader et al., 2012). The combination of negative NTCs, proportional input response, and non-amplifications across triplicates indicates viral levels are below the detection limit. Our findings revealed cultivar-specific responses to the various virus elimination treatments tested, aligning with some previous research, while adding new insights into the application of oseltamivir as a plant viricide and refined cryotherapy protocols.

To improve shoot tip survival, we incorporated an antioxidant-enriched preculture medium containing SA, ascorbic acid, and reduced glutathione. This approach contrasts with earlier protocols that relied primarily on osmotic preconditioning (Wang et al., 2003; Bi et al., 2018) and represents a significant advancement in cryopreservation techniques for grapevine virus eradication (Pathirana et al., 2013, 2016; Bettoni et al., 2021). The combination of thermotherapy and cryotherapy resulted in the highest rates of GLRaV-3 eradication, achieving 100% virus elimination in Sauvignon Blanc 217, Chenin Blanc, and Riesling Gm 239. These results are consistent with earlier studies highlighting the effectiveness of cryotherapy for the elimination of GLRaV-3 and other grapevine viruses (Pathirana et al., 2013; Bi et al., 2018; Wang et al., 2003; Marković et al., 2015).

Pathirana et al. (2016), Bi et al. (2018), and Marković et al. (2015) reported high success rates using shoot tip cryotherapy for GLRaV-3 eradication, with Bi et al. (2018) emphasising the critical importance of shoot tip size and meristematic cell survival during the freezing process. Our findings reinforce the value of using shoot tips and further demonstrate that including both apical and axillary shoot tips can enhance survival and regeneration rates in certain cultivars, particularly Chenin Blanc. Nevertheless, variable responses among cultivars to the treatment conditions were observed. For example, Golden Chasselas exhibited reduced plantlet regeneration, reflecting the variability previously reported in grapevine cryotherapy studies (Marković et al., 2015; Wang and Valkonen, 2009).

The use of oseltamivir, a neuraminidase inhibitor commonly employed for human influenza treatment (Moscona, 2005), was previously evaluated by Guţă et al. (2014) in combination with ribavirin for the elimination of grapevine fleck virus. To our knowledge, this is the first report of oseltamivir used in combination with thermotherapy for the elimination of GLRaV-3 in multiple grapevine cultivars. The approach evaluated here achieved GLRaV-3 virus elimination to below the limit of detection in Ehrenfelser and Golden Chasselas, and partial success in Chenin Blanc and Sauvignon Blanc 217.

The use of oseltamivir in combination with thermotherapy resulted in complete elimination of the GLRaV-3 in certain cultivars, surpassing conventional ribavirin-based virus treatments. Although we used the upper-limit dose (25 μg mL^-^¹) reported by Hu et al. (2020), the superior performance of the oseltamivir regimen over ribavirin in our study may reflect differences in antiviral mechanism. Ribavirin induces lethal mutagenesis of viral RNA (Crotty et al., 2002), and oseltamivir may exert broader inhibitory effects on GLRaV-3 replication in plants, as the precise mechanism remains to be elucidated. Phytotoxic effects resulting from chemotherapy-based treatments, including oseltamivir, were reported by Guţă et al. (2014) in experimental plant cultures. Notably, in our study, the addition of 10 μM SA in the oseltamivir-based treatment protocol could have contributed to enhanced plant defence responses, as SA is known to stimulate systemic acquired resistance and bolster innate immunity against viral infections (Vlot et al., 2009; Pathirana et al., 2016). Consistent with Hu et al. (2020), our ribavirin regimen did not include SA in the thermotherapy medium. Because SA was not incorporated into the ribavirin treatment medium, we are unable to quantify any SA-specific effects on antiviral efficacy or phytotoxicity. Our findings suggest that oseltamivir, when combined with SA, could offer a promising alternative to ribavirin for GLRaV-3 virus elimination, particularly considering ribavirin’s inconsistent success across various grapevine cultivars (Skiada et al., 2013; Hu et al., 2018). In contrast, the combination of ribavirin-based chemotherapy with thermotherapy produced the fewest GLRaV-3 virus free plantlets among the techniques tested. While partial GLRaV-3 virus elimination was achieved in Ehrenfelser and Chenin Blanc, no virus-free plantlets were regenerated from Sauvignon Blanc 217, Riesling Gm 239, or Golden Chasselas. These findings are consistent with previous reports noting that ribavirin’s efficiency is highly variable depending on grapevine cultivar, virus strain, and treatment conditions (Hu et al., 2021; Panattoni et al., 2013). Moreover, the limited survival and poor regeneration observed following ribavirin treatment underscore a significant constraint that could compromise the scalability and consistency of its application in grapevine germplasm sanitation initiatives. From a practical standpoint, combining thermotherapy with chemotherapy (ribavirin and oseltamivir) differs significantly from thermotherapy followed by cryotherapy, particularly regarding labour intensity, duration, and required technical expertise. The combination of thermotherapy and chemotherapy involves simple, sequential procedures and requires minimal specialised equipment or reagents, supporting its feasibility and cost-effectiveness. However, this approach demands careful monitoring of antiviral compound concentrations to prevent phytotoxicity, particularly during prolonged heat treatments. Consistent with this, Wang et al. (2018) reported similar findings, observing higher plant recovery rates with combined thermotherapy and chemotherapy compared to cryotherapy-based treatments, although the virus elimination efficiency was somewhat lower. In contrast, thermotherapy followed by cryotherapy typically achieves higher virus eradication rates but requires specialised cryoprotectants and meticulous handling of apical shoot tips before, during, and after exposure to liquid nitrogen (Zhao et al., 2018). Consequently, this technique is more labour-intensive, demands greater technical skill, and generally requires more time than the combined thermotherapy and chemotherapy approach. Although thermotherapy followed by cryotherapy achieved 100% virus eradication in this study, it resulted in significantly lower shoot regrowth compared to the combination of thermotherapy and chemotherapy, consistent with the findings of Bettoni et al. (2021), who also reported this eradication–regrowth trade-off. Nevertheless, it is important to note that outcomes may vary depending on plant species, virus strains, and specific procedural optimisations.

Our findings also underscore the critical value of combining sensitive diagnostic methods such as FPST RT-qPCR for accurate virus detection across the various treated plantlets. FPST RT-qPCR, targeting the conserved 3′ untranslated region of GLRaV-3, allowed for the sensitive detection of divergent viral strains, as previously demonstrated by Diaz-Lara et al. (2018). This diagnostic approach ensures more reliable verification of GLRaV-3 virus-free status, a factor increasingly emphasised in grapevine sanitation and certification programmes (Villamor et al., 2019).

In conclusion, our study demonstrates that thermotherapy combined with cryotherapy offers an effective and widely applicable method for GLRaV-3 eradication across diverse grapevine genotypes. Virus-free plants produced by this approach have broad practical value, including for the reliable supply of GLRaV-3 virus-free material for establishing new vineyards, supporting grapevine certification programs, building high-health germplasm repositories in the field and via *in vitro* technologies such as slow-growth and cryopreserved collections, and facilitating regional and international germplasm exchange. Meanwhile, oseltamivir combined with thermotherapy shows significant promise as an antiviral approach, outperforming ribavirin in several cases. Hence, future work should focus on exploring the mechanistic action of oseltamivir in plant systems. These advances will contribute to safeguarding valuable germplasm collections and supporting the long-term sustainability of the viticulture industry in New Zealand and globally.

## Data Availability

The data supporting the findings of this study have been deposited in NCBI under BioProject PRJNA1347987. Associated BioSamples are: SAMN52879009 : Sample_12_6 (TaxID: 29760), SAMN52879010 : Sample_10_43 (TaxID: 29760), SAMN52879011 : Sample_10_31 (TaxID: 29760), SAMN52879012 : Sample_11_109 (TaxID: 29760), SAMN52879013 : Sample_10_86 (TaxID: 29760).

## Supplementary material

Supplementary Figure 1 FPST RT-qPCR detection of GLRaV-3 in pre-treated grapevine cultivars using 10 and 5 ng/µL RNA templates (each in triplicate). Positive amplification was obtained at both inputs, with 5 ng/µL yielding GLRaV-3 detection across all cultivars.

Supplementary Figure 2 FPST qRT-PCR amplification of GLRaV-3 using non-treated Sauvignon Blanc 217 RNA (159 ng/µL) and a 10-fold dilution series (8, 0.8, 0.08 ng/µL), each run in duplicate. The lowest concentration (0.08 ng/µL) also amplified, indicating a positive GLRaV-3 result at this dilution.

Supplementary Figure 3 FPST RT-qPCR amplification plots for GLRaV-3 from lines treated with thermotherapy followed by cryotherapy or with combined thermotherapy–chemotherapy (oseltamivir) in Sauvignon Blanc 217, Ehrenfelser, and Riesling Gm 239. Reactions were performed in triplicate with ≥10 ng total RNA per reaction. No amplification was detected by cycle 40 in treated lines across cultivars, consistent with the predefined criteria for a negative GLRaV-3 call.

## References

[B1] Adil S. Singh V. Anjum A. Quraishi A. (2022). A mini-review on electrotherapeutic strategy for the plant viral elimination. Plant Cell. Tissue Organ Cult. (PCTOC). 150, 41–55. doi: 10.1007/s11240-022-02265-w

[B2] Alleweldt G. (1994). “ World list of grapevine collections,” in Bundesanstalt für Züchtungsforschung an Kulturpflanzen (Siebeldingen, Germany: Institut für Rebenzüchtung Geilweilerhof).

[B3] Bell V. A. Lester P. J. Pietersen G. Hall A. J. (2021). The management and financial implications of variable responses to grapevine leafroll disease. J. Plant Pathol. 103, 5–15. doi: 10.1007/s42161-020-00736-7

[B4] Bettoni J. Bonnart R. Volk G. M. (2020). “ Vitis shoot tip cryopreservation (Droplet vitrification and V-cryoplate),” in Training in Plant Genetic Resources: Cryopreservation of Clonal Propagules. Ed. Volk G. M. ( Colorado State University, Fort Collins, Colorado). Available online at: https://colostate.pressbooks.pub/clonalcryopreservation/chapter/vitis-shoot-tip-cryopreservation-droplet-vitrification-and-cryoplate/ (Accessed December 15, 2025).

[B5] Bettoni J. C. Costa M. D. Gardin J. P. P. Kretzschmar A. A. Pathirana R. (2016). Cryotherapy: a new technique to obtain grapevine plants free of viruses. Rev. Bras. Fruticult. 38, e–833. doi: 10.1590/0100-29452016833

[B6] Bettoni J. C. Marković Z. Bi W. Volk G. M. Matsumoto T. Wang Q. C. (2021). Grapevine shoot tip cryopreservation and cryotherapy: Secure storage of disease-free plants. Plants 10, 2190. doi: 10.3390/plants10102190, PMID: 34685999 PMC8541583

[B7] Bettoni J. C. Mathew L. Pathirana R. Wiedow C. Hunter D. A. McLachlan A. . (2022). Eradication of *Potato Virus S*, *Potato Virus A*, and *Potato Virus M* From infected *in vitro*-grown potato shoots using *in vitro* therapies. Front. Plant Sci. 13. doi: 10.3389/fpls.2022.878733, PMID: 35665190 PMC9161163

[B8] Bi W. L. Hao X. Y. Cui Z. H. Pathirana R. Volk G. M. Wang Q. C. (2018). Shoot tip cryotherapy for efficient eradication of grapevine leafroll-associated virus-3 from diseased grapevine *in vitro* plants. Ann. Appl. Biol. 173, 261–270. doi: 10.1111/aab.12459

[B9] Bolger A. M. Lohse M. Usadel B. (2014). Trimmomatic: a flexible trimmer for Illumina sequence data. Bioinformatics 30, 2114–2120. doi: 10.1093/bioinformatics/btu170, PMID: 24695404 PMC4103590

[B10] Bustin S. A. Benes V. Garson J. A. Hellemans J. Huggett J. Kubista M. . (2009). The MIQE guidelines: minimum information for publication of quantitative real-time PCR experiments. Clin. Chem. 55, 611–622. doi: 10.1373/clinchem.2008.112797, PMID: 19246619

[B11] Candresse T. Faure C. Marais A. (2023). First report of grapevine red globe virus (GRGV) and grapevine rupestris vein feathering virus (GRVFV) infecting grapevine (Vitis vinifera) in Portugal. Plant Dis. 107, 974. doi: 10.1094/PDIS-06-22-1326-PDN

[B12] Chooi K. M. Bell V. A. Blouin A. G. Cohen D. Mundy D. Henshall W. . (2022). Grapevine leafroll-associated virus 3 genotype influences foliar symptom development in New Zealand vineyards. Viruses 14, 1348. doi: 10.3390/v14071348, PMID: 35891330 PMC9316759

[B13] Chooi K. M. Bell V. A. Blouin A. G. Sandanayaka M. Gough R. Chhagan A. . (2024). The New Zealand perspective of an ecosystem biology response to grapevine leafroll disease. Adv. Virus Res. 118, 213–272. doi: 10.1016/bs.aivir.2024.02.001, PMID: 38461030

[B14] Crotty S. Cameron C. Andino R. (2002). Ribavirin’s antiviral mechanism of action: Lethal mutagenesis? J. Mol. Med. 80, 86–95. doi: 10.1007/s00109-001-0308-0, PMID: 11907645

[B15] Diaz-Lara A. Klaassen V. Stevens K. Sudarshana M. R. Rowhani A. Maree H. J. . (2018). Characterization of grapevine leafroll-associated virus 3 genetic variants and application towards RT-qPCR assay design. PloS One 13, e0208862. doi: 10.1371/journal.pone.0208862, PMID: 30540844 PMC6291115

[B16] Fuchs M. (2020). Grapevine viruses: A multitude of diverse species with simple but overall poorly adopted management solutions in the vineyard. J. Plant Pathol. 102, 643–653. doi: 10.1007/s42161-020-00579-2

[B17] Guţă I. C. Buciumeanu E. C. Vişoiu E. (2014). Elimination of Grapevine fleck virus by *in vitro* Chemotherapy. Notulae. Bot. Horti. Agrobot. Cluj-Napoca. 42, 115–118. doi: 10.15835/nbha4219227

[B18] Hu G. J. Dong Y. F. Zhang Z. P. Fan X. D. Fang R. E. N. (2021). Elimination of grapevine fleck virus and grapevine rupestris stem pitting-associated virus from Vitis vinifera 87–1 by ribavirin combined with thermotherapy. J. Integr. Agric. 20, 2463–2470. doi: 10.1016/S2095-3119(20)63336-6

[B19] Hu G. Dong Y. Zhang Z. Fan X. Ren F. (2020). Efficiency of chemotherapy combined with thermotherapy for eliminating grapevine leafroll-associated virus 3 (GLRaV-3). Sci. Hortic. 271, 109462. doi: 10.1016/j.scienta.2020.109462

[B20] Hu G. Dong Y. Zhang Z. Fan X. Ren F. Li Z. . (2018). Elimination of grapevine rupestris stem pitting-associated virus from Vitis vinifera ‘Kyoho’ by an antiviral agent combined with shoot tip culture. Sci. Hortic. 229, 99–106. doi: 10.1016/j.scienta.2017.10.041

[B21] Kopylova E. Noé L. Touzet H. (2012). SortMeRNA: fast and accurate filtering of ribosomal RNAs in metatranscriptomic data. Bioinformatics 28, 3211–3217. doi: 10.1093/bioinformatics/bts611, PMID: 23071270

[B22] Kotni P. van Hintum T. Maggioni L. Oppermann M. Weise S. (2023). EURISCO update 2023: the European Search Catalogue for Plant Genetic Resources, a pillar for documentation of genebank material. Nucleic Acids Res. 51, D1465–D1469. doi: 10.1093/nar/gkac852, PMID: 36189883 PMC9825528

[B23] Liu J. Zhang X. Yang Y. Hong N. Wang G. Wang A. . (2016). Characterisation of virus-derived small interfering RNAs in Apple stem grooving virus-infected *in vitro*-cultured Pyrus pyrifolia shoot tips in response to high temperature treatment. Virol. J. 13, 166. doi: 10.1186/s12985-016-0625-0, PMID: 27716257 PMC5053029

[B24] Liu J. Zhang X. Zhang F. Hong N. Wang G. Wang A. . (2015). Identification and characterisation of microRNAs from *in vitro*-grown pear shoots infected with Apple stem grooving virus in response to high temperature using small RNA sequencing. BMC Genomics 16, 945. doi: 10.1186/s12864-015-2126-8, PMID: 26573813 PMC4647338

[B25] Magon G. De Rosa V. Martina M. Falchi R. Acquadro A. Barcaccia G. . (2023). Boosting grapevine breeding for climate-smart viticulture: from genetic resources to predictive genomics. Front. Plant Sci. 14, 1293186. doi: 10.3389/fpls.2023.1293186, PMID: 38148866 PMC10750425

[B26] Magyar-Tábori K. Mendler-Drienyovszki N. Hanász A. Zsombik L. Dobránszki J. (2021). Phytotoxicity and other adverse effects on the *in vitro* shoot cultures caused by virus elimination treatments: reasons and solutions. Plants 10, 670. doi: 10.3390/plants10040670, PMID: 33807286 PMC8066107

[B27] Maliogka V. I. Martelli G. P. Fuchs M. Katis N. I. (2015). Control of viruses infecting grapevine. Adv. Virus Res. 91, 175–227. doi: 10.1016/bs.aivir.2014.11.002, PMID: 25591880

[B28] Maliogka V. I. Skiada F. G. Eleftheriou E. P. Katis N. I. (2009). Elimination of a new ampelovirus (GLRaV-Pr) and Grapevine rupestris stem pitting associated virus (GRSPaV) from two Vitis vinifera cultivars combining *in vitro* thermotherapy with shoot tip culture. Sci. Hortic. 123, 280–282. doi: 10.1016/j.scienta.2009.08.016

[B29] Marković Z. Preiner D. Stupić D. Andabaka Ž. Šimon S. Vončina D. . (2015). Cryopreservation and cryotherapy of grapevine (*Vitis vinifera* L.). Vitis – J. Grapevine. Res. 54, 247–251.

[B30] Mathew L. Tiffin H. Erridge Z. McLachlan A. Hunter D. Pathirana R. (2021). Efficiency of eradication of Raspberry bushy dwarf virus from infected raspberry (Rubus idaeus) by *in vitro* chemotherapy, thermotherapy and cryotherapy and their combinations. Plant Cell. Tissue Organ Cult. (PCTOC). 144, 133–141. doi: 10.1007/s11240-020-01829-y

[B31] Maul E. This P. (2008). GENRES 081-a basis for the preservation and utilization of Vitis genetic resources. Rome: Bioversity International.

[B32] Miljanić V. Rusjan D. Škvarč A. Chatelet P. Štajner N. (2022). Elimination of eight viruses and two viroids from preclonal candidates of six grapevine varieties (*Vitis vinifera* L.) through *in vivo* thermotherapy and *in vitro* meristem tip micrografting. Plants 11, 1064., PMID: 35448791 10.3390/plants11081064 PMC9029751

[B33] Moscona A. (2005). Neuraminidase inhibitors for influenza. New Engl. J. Med. 353, 1363–1373. doi: 10.1056/NEJMra050740, PMID: 16192481

[B34] Murashige T. Skoog F. (1962). A revised medium for rapid growth and bio assays with tobacco tissue cultures. Physiol. Plant. 15, 473–497. doi: 10.1111/j.1399-3054.1962.tb08052.x

[B35] National Academies of Sciences, Engineering, and Medicine (2025). Advancing vineyard Health: Insights and Innovations for Combating Grapevine Red Blotch and Leafroll Diseases (Washington, DC, United States of America: The National Academies Press). doi: 10.17226/27472

[B36] Panattoni A. Luvisi A. Triolo E. (2013). Elimination of viruses in plants: twenty years of progress. Spanish. J. Agric. Res. 11, 173–188. doi: 10.5424/sjar/2013111-3201

[B37] Pathirana R. McLachlan A. Hedderley D. Carra A. Carimi F. Panis B. (2013). “ Removal of leafroll viruses from infected grapevine plants by droplet vitrification,” in VIII International Symposium on In Vitro Culture and Horticultural Breeding 1083. *Proceedings of the VIIIth InternaAonal Symposium on In Vitro Culture and HorAcultural Breeding: Coimbra, Portugal, June 2–7, 2013* (Acta Hor-culturae No. 1083). Interna-onal Society for Hor-cultural Science (ISHS). 491–498.

[B38] Pathirana R. McLachlan A. Hedderley D. Panis B. Carimi F. (2016). Pre-treatment with salicylic acid improves plant regeneration after cryopreservation of grapevine (*Vitis* spp.) by droplet vitrification. Acta Physiol. Plant. 38, 12. doi: 10.1007/s11738-015-2026-1

[B39] Prjibelski A. Antipov D. Meleshko D. Lapidus A. Korobeynikov A. (2020). Using SPAdes *de novo* assembler. Curr. Protoc. Bioinf. 70, e102. doi: 10.1002/cpbi.102, PMID: 32559359

[B40] Sakai A. Kobayashi S. Oiyama I. (1990). Cryopreservation of nucellar cells of navel orange (Citrus sinensis Osb. var. brasiliensis Tanaka) by vitrification. Plant Cell Rep. 9, 30–33. doi: 10.1007/BF00232130, PMID: 24226373

[B41] Schrader C. Schielke A. Ellerbroek L. Johne R. (2012). PCR inhibitors–occurrence, properties and removal. J. Appl. Microbiol. 113, 1014–1026. doi: 10.1111/j.1365-2672.2012.05384.x, PMID: 22747964

[B42] Sharma A. M. Wang J. Duffy S. Zhang S. Wong M. K. Rashed A. . (2011). Occurrence of grapevine leafroll-associated virus complex in Napa Valley. PloS One 6, e26227. doi: 10.1371/journal.pone.0026227, PMID: 22039446 PMC3198396

[B43] Skiada F. G. Maliogka V. I. Katis N. I. Eleftheriou E. P. (2013). Elimination of Grapevine rupestris stem pitting-associated virus (GRSPaV) from two Vitis vinifera cultivars by *in vitro* chemotherapy. Eur. J. Plant Pathol. 135, 407–414. doi: 10.1007/s10658-012-0097-z

[B44] Spiegel S. Frison E. A. Converse R. H. (1993). Recent developments in therapy and virus-detection procedures for international movement of clonal plant germplasm. Plant Dis. 77, 1176–1180. doi: 10.1094/PD-77-1176

[B45] Szabó L. K. Desiderio F. Kirilla Z. Hegedűs A. Várallyay É. Preininger É. (2024). A mini-review on *in vitro* methods for virus elimination from Prunus sp. fruit trees. Plant Cell. Tissue Organ Cult. 156, 42. doi: 10.1007/s11240-023-02670-9

[B46] Szittya G. Silhavy D. Molnár A. Havelda Z. Lovas Á. Lakatos L. . (2003). Low temperature inhibits RNA silencing-mediated defence by the control of siRNA generation. EMBO J. 22, 633–640. doi: 10.1093/emboj/cdg74, PMID: 12554663 PMC140757

[B47] Turcsan M. Demian E. Varga T. Jaksa-Czotter N. Szegedi E. Olah R. . (2020). Hts-based monitoring of the efficiency of somatic embryogenesis and meristem cultures used for virus elimination in grapevine. Plants 9, 1782. doi: 10.3390/plants9121782, PMID: 33339181 PMC7765609

[B48] Villamor D. E. V. Ho T. Al Rwahnih M. Martin R. R. Tzanetakis I. E. (2019). High throughput sequencing for plant virus detection and discovery. Phytopathology 109, 716–725. doi: 10.1094/PHYTO-07-18-0257-RVW, PMID: 30801236

[B49] Vlot A. C. Dempsey D. M. A. Klessig D. F. (2009). Salicylic acid, a multifaceted hormone to combat disease. Annu. Rev. Phytopathol. 47, 177–206. doi: 10.1146/annurev.phyto.050908.135202, PMID: 19400653

[B53] Wang Q. Cuellar W. J. Rajamäki M. L. Hirata Y. Valkonen J. P. (2008). Combined thermotherapy and cryotherapy for efficient virus eradication: relation of virus distribution, subcellular changes, cell survival and viral RNA degradation in shoot tips. Mol. Plant Pathol. 9, 237–250. doi: 10.1111/j.1364-3703.2007.00456.x, PMID: 18705855 PMC6640318

[B50] Wang M. R. Cui Z. H. Li J. W. Hao X. Y. Zhao L. Wang Q. C. (2018). *In vitro* thermotherapy-based methods for plant virus eradication. Plant Methods 14, 87. doi: 10.1186/s13007-018-0355-y, PMID: 30323856 PMC6173849

[B51] Wang M. R. Li B. Q. Feng C. H. Wang Q. C. (2016). Culture of shoot tips from adventitious shoots can eradicate Apple stem pitting virus but fails in Apple stem grooving virus. Plant Cell. Tissue Organ Cult. (PCTOC). 125, 283–291. doi: 10.1007/s11240-016-0948-y

[B54] Wang Q. Mawassi M. Li P. Gafny R. Sela I. Tanne E. (2003). Elimination of grapevine virus A (GVA) by cryopreservation of *in vitro*-grown shoot tips of Vitis vinifera L. Plant Sci. 165, 321–327. doi: 10.1016/S0168-9452(03)00091-8

[B52] Wang Q. Valkonen J. P. (2009). Cryotherapy of shoot tips: novel pathogen eradication method. Trends Plant Sci. 14, 119–122. doi: 10.1016/j.tplants.2008.11.010, PMID: 19217342

[B55] Wante S. P. Leung D. W. Alizadeh H. (2022). Sub-lethal effect of diesel fuel on the development of *in vitro* produced callus culture of *Tagetes patula* L. (Marigold–Nemo mix). Vegetos 35, 1118–1127. doi: 10.1007/s42535-022-00390-7

[B5000] Wijerathna-Yapa A. Hi-Bandaralage J. Pathirana R. (2025). Harnessing metabolites from plant cell tissue and organ culture for sustainable biotechnology. Plant Cell, Tissue and Organ Culture (PCTOC). 62 (3), 55. doi: 10.1007/s11240-025-03180-6

[B56] Xiao H. Meng B. (2023). Molecular and metagenomic analyses reveal high prevalence and complexity of viral infections in french-american hybrids and north american grapes. Viruses 15, 1949. doi: 10.3390/v15091949, PMID: 37766355 PMC10534776

[B57] Zhao L. Wang M. R. Cui Z. H. Chen L. Volk G. M. Wang Q. C. (2018). Combining thermotherapy with cryotherapy for efficient eradication of Apple stem grooving virus from infected *in-vitro*-cultured apple shoots. Plant Dis. 102, 1574–1580. doi: 10.1094/PDIS-11-17-1753-RE, PMID: 30673422

