# Supplementary figures and images for: Assessing the efficacy of thermotherapy combined with chemotherapy or cryotherapy for the eradication of grapevine leafroll-associated virus 3
Source: Front Plant Sci. 2026 Jan 5;16:1693493. doi: 10.3389/fpls.2025.1693493 (PMC12826072; doi:10.3389/fpls.2025.1693493)

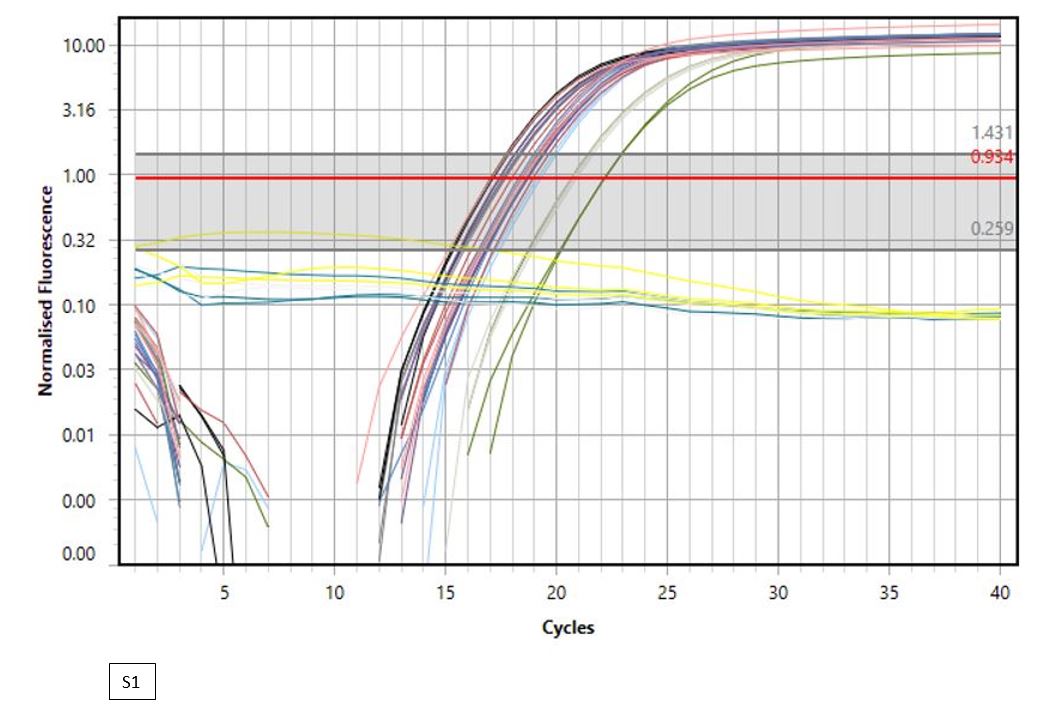

Supplement: Supplementary Figure 1 — FPST RT-qPCR detection of GLRaV-3 in pre-treated grapevine cultivars using 10 and 5 ng/µL RNA templates (each in triplicate). Positive amplification was obtained at both inputs, with 5 ng/µL yielding GLRaV-3 detection across all cultivars. [file Image1.jpeg]

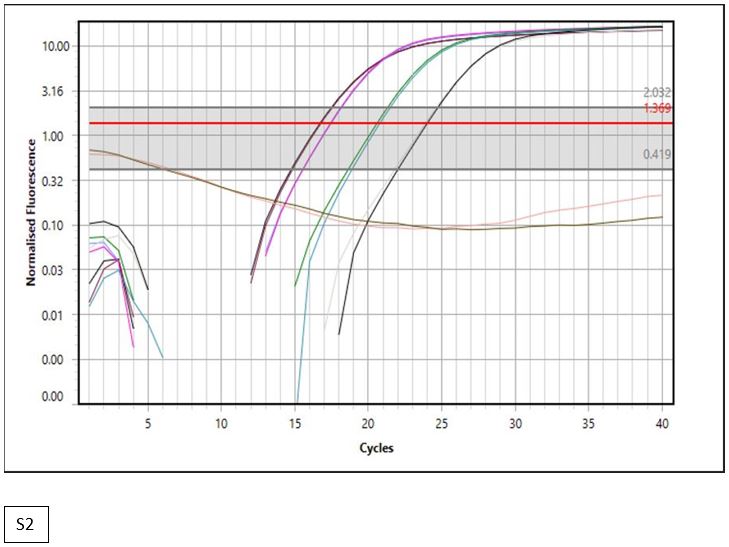

Supplement: Supplementary Figure 2 — FPST qRT-PCR amplification of GLRaV-3 using non-treated Sauvignon Blanc 217 RNA (159 ng/µL) and a 10-fold dilution series (8, 0.8, 0.08 ng/µL), each run in duplicate. The lowest concentration (0.08 ng/µL) also amplified, indicating a positive GLRaV-3 result at this dilution. [file Image2.jpeg]

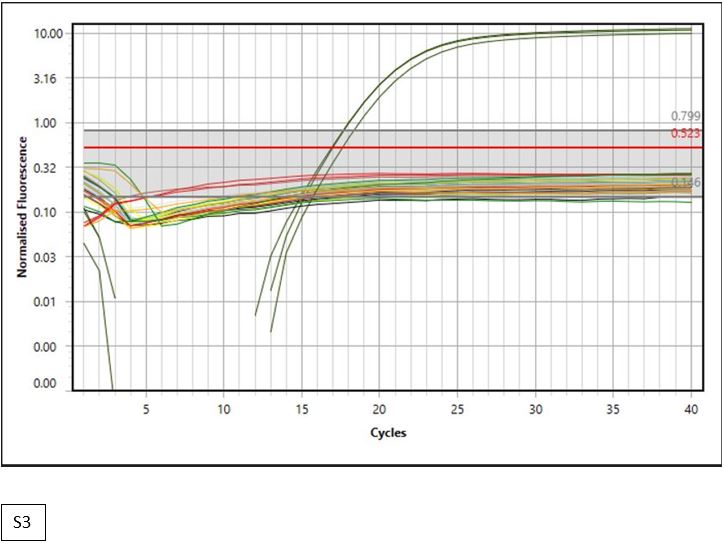

Supplement: Supplementary Figure 3 — FPST RT-qPCR amplification plots for GLRaV-3 from lines treated with thermotherapy followed by cryotherapy or with combined thermotherapy–chemotherapy (oseltamivir) in Sauvignon Blanc 217, Ehrenfelser, and Riesling Gm 239. Reactions were performed in triplicate with ≥10 ng total RNA per reaction. No amplification was detected by cycle 40 in treated lines across cultivars, consistent with the predefined criteria for a negative GLRaV-3 call. [file Image3.jpeg]
